# Epicardium-Derived Tbx18^+^ CDCs Transplantation Improve Heart Function in Infarcted Mice

**DOI:** 10.3389/fcvm.2021.744353

**Published:** 2022-01-24

**Authors:** Zhenglong Guo, Mengyuan Geng, Litao Qin, Bingtao Hao, Shixiu Liao

**Affiliations:** ^1^Henan Medical Genetics Institute, Henan Provincial Key Laboratory of Genetic Diseases and Functional Genomics, National Health Commission Key Laboratory of Birth Defects Prevention, People's Hospital of Zhengzhou University, Henan Provincial People's Hospital, Zhengzhou, China; ^2^School of Medical Laboratory and Tianjin Key Laboratory of Cellular Homeostasis and Human Diseases, Tianjin Medical University, Tianjin, China; ^3^School of Basic Medical Sciences, Cancer Research Institute, Southern Medical University, Guangzhou, China

**Keywords:** epicardium, CDCs, epicardial cells, Tbx18, hTERT, myocardial infarction

## Abstract

Cardiosphere-derived cells (CDCs) constitute a cardiac stem cell pool, a promising therapeutics in treating myocardial infarction (MI). However, the cell source of CDCs remains unclear. In this study, we isolated CDCs directly from adult mouse heart epicardium named primary epicardium-derived CDCs (pECDCs), which showed a different expression profile compared with primary epicardial cells (pEpiCs). Interestingly, pECDCs highly expressed T-box transcription factor 18 (Tbx18) and showed multipotent differentiation ability *in vitro*. Human telomerase reverse transcriptase (hTERT) transduction could inhibit aging-induced pECDCs apoptosis and differentiation, thus keeping a better proliferation capacity. Furthermore, immortalized epicardium CDCs (iECDCs) transplantation extensively promote cardiogenesis in the infracted mouse heart. This study demonstrated epicardium-derived CDCs that may derive from Tbx18^+^ EpiCs, which possess the therapeutic potential to be applied to cardiac repair and regeneration and suggest a new kind of CDCs with identified origination that may be followed in the developing and injured heart.

## Introduction

Cardiosphere-derived cells (CDCs) are a heterogeneous mixture of cardiac cell pools, including cardiac progenitor cells (CPCs), derived from heart biopsy culture (1, 2). Messina et al. firstly illustrated the isolation and expansion method of adult cardiac stem cells from human and murine heart (3). Antigenic profiles revealed that CDCs are positive of CD105 and CD90 and are negative of hematopoietic cell and endothelial cell markers, CD31 and CD45 (2, 4, 5). CDCs possess a multipotential differentiation ability *in vitro*, mainly for cardiomyocytes, endothelial cells, and smooth muscle cells (1, 6). In the recent years, CDCs transplantation and paracrine exosomes emerged as a promising therapeutic option for many cardiac diseases including myocardial infarction (MI) (7, 8), dilated cardiomyopathy (9, 10), and dystrophic cardiomyopathy (11, 12). Many clinical trials have proved the safety and efficiency of autologous and allogeneic CDCs therapy such as the CArdiosphere-Derived aUtologous stem CElls to Reverse ventricUlar dySfunction (CADUCEUS) trial (13), the AutoLogous Human CArdiac-Derived Stem Cell to Treat Ischemic cArdiomyopathy (ALCADIA) trial (14), the ALLogeneic Heart STem Cells to Achieve Myocardial Regeneration (ALLSTAR) trial (15), and the Dilated cardiomYopathy iNtervention with Allogeneic MyocardIally-regenerative Cells (DYNAMIC) trial (16). Moreover, the Halt cardiomyOPathy progrEssion (HOPE)-Duchenne trial assessed feasibility, safety, and efficacy of intracoronary allogeneic CDCs (CAP-1002) in patients with Duchenne muscular dystrophy (DMD) (17). Although accumulating evidence demonstrated that CDCs graft contributed to the heart function improvement and inflammation inhibition (18–20) and little is known about origin of these cells. Conventional CDCs from heart biopsy are composed of a mixture of epicardium-, myocardium-, and endocardium-derived subpopulations. Epicardial cells (EpiCs) or epicardium-derived cells (EPDCs) are of vital importance to heart development and regeneration (21–24), which mostly owe to the epithelial–mesenchymal transition (EMT) migration and differentiation regulated through T-box transcription factor 18 (Tbx18) and Wilms' tumor suppressor transcription factor (Wt1) (25–30). Up to now, whether epicardium could yield functional CDCs remains unclear.

In this study, we first isolated and identified adult heart epicardium-derived CDCs-epicardium CDCs (ECDCs), which exhibit a cardiac gene expression profile, especially for Tbx18. Tbx18-expressing EpiCs were demonstrated as progenitor cells giving rise to multiple cardiovascular cell types during heart development (31). Correspondingly, Tbx18-expressing ECDCs showed multipotent differentiation ability *in vitro* and immortalized epicardium CDCs (iECDCs) transplantation significantly promoted cardiogenesis and improved left ventricular function in the failing heart following MI in mice, which provide a method to isolate epicardial Tbx18^+^ cells and offer a candidate cell type for cell therapy in cardiovascular diseases.

## Materials and Methods

### Primary Epicardium-Derived CDCs (pECDCs) Isolation and Culture

Primary epicardium-derived CDCs were generated from 6-week-old C56BL/6 mouse epicardium as described in the article with some modifications (32). In detail, the pericardial parietal layer, which was attached to diaphragm, sternum, and anterior mediastinum, was firstly peeled off. Then, the heart was removed and the epicardium was gently isolated piece by piece with the careful usage of forceps, due to high difficulty in complete stripping off the epicardium. Subsequently, the ventricle epicardium was cut into fragments <1 mm^3^ in the worktable, following by wash with sterile phosphate-buffered saline (PBS) and partially digested with 0.05% trypsin. Then, these fragments were seeded on fibronectin (Sigma-Aldrich, St.Louis, MO, USA) (20 μg/ml) coated dishes in complete explant medium (CEM) [Iscove's Modified Dulbecco's Medium (IMDM) (Invitrogen, Carlsbad, CA, USA), 20% fetal bovine serum (FBS) (Invitrogen, Carlsbad, CA, USA), 1% penicillin/streptomycin (P/S) (Invitrogen, Carlsbad, CA, USA), 1% Glutamax (Invitrogen, Carlsbad, CA, USA), and 0.1 mM β-mercaptoethanol (Sigma-Aldrich, St.Louis, MO, USA)]. During the first week of growth, a layer of stromal-like cells emerge from adherent explants over which small, round, and phase-bright cells migrated. Once the cells surrounding the explant were confluent, 0.5 mM Ethylene Diamine Tetraacetic Acid (EDTA) and 0.025% trypsin digestion were used to harvest these cells. Explant outgrowth could be harvested up to four times from the same specimen. These cells were seeded at 2 × 10^4^ cells/ml on poly-D-lysine (40 μg/ml) coated dishes in cardiosphere-growing medium (CGM) [35% IMDM/65% Dulbecco's modified Eagle's medium (DMEM)-Ham's F-12 (Invitrogen, Carlsbad, CA, USA), 5% FBS, 1% P/S, 1% Glutamax, 0.1 mM β-mercaptoethanol, 2% B27 (Invitrogen, Carlsbad, CA, USA), 1 U/ml thrombin (Invitrogen, Carlsbad, CA, USA), 80 ng/ml basic fibroblast growth factor (bFGF) (PeproTech, Cranbury, NJ, USA), 25 ng/ml Epidermal Growth Factor (EGF) (PeproTech, Cranbury, NJ, USA), and 4 ng/ml cardiotrophin-1 (PeproTech, Cranbury, NJ, USA)], in which ECDCs could form cardiospheres. Four weeks later, cells that remained adherent to the dishes were discarded, whereas detached cardiospheres were collected and plated on fibronectin-coated flasks and expanded as fibroblast-like cells. ECDCs were subsequently passaged by 0.25% trypsin and cultured in CEM. Primary ECDCs at passage 3 were used for subsequent experiments.

### Isolation of Primary EpiCs

For the isolation of EpiCs, we followed the method referred by Zhou and Pu (33). Briefly, the heart was dissected from 6 to 8-week-old C57BL/6 and rinsed in D-Hanks' buffer and digested with sterilized D-Hanks' buffer supplemented with 0.08% collagenase IV (Worthington, Lakewood, NJ, USA) and 0.05% trypsin (Gibco, Carlsbad, CA, USA) at 37°C for 8 min under gentle rotation (60 rpm/min) and repeated for 8 times. Subsequently, cells were centrifuged at 1,000 rpm for 5 min and seeded onto 0.1% gelatin-coated 24-well plates with culture medium containing low glucose DMEM (Invitrogen, Carlsbad, CA, USA) and Medium 199 (M199) (Invitrogen, Carlsbad, CA, USA) in a 1:1 ratio, supplemented with 10% heat-inactivated (56°C for 25 min) FBS (Gibco, Carlsbad, CA, USA) and 1% P/S (Invitrogen, Carlsbad, CA, USA). Primary EpiCs at passage 3 were used for subsequent experiments.

### Lentivirus Preparation and Infection

Lentivirus was prepared with three plasmids (40 μg in total), including the packaging plasmids psPAX2 and pM2D.G and human telomerase reverse transcriptase (hTERT) (plenti-CMV-hTERT, ABM, Vancouver, Canada), mixed in a ratio of 3:1:4 and 293FT cells were transiently transfected using calcium phosphate method. Viral supernatant from transfected 10 cm dish was collected every 24 h up to 2 days after the transfection. For hTERT-expressing lentivirus infection, ECDCs (passage 1, 5–10 × 10^5^) were seeded in 6-well plates and infected with 1.5 ml viral supernatant mixed with 0.5 ml fresh CEM containing 1/1,000 polybrene for 12 h and then replaced with fresh CEM. 1 week later, these cells were selected by 2 μg/ml puromycin for 2 weeks.

### Karyotype and Telomere Analysis

The karyotype of pECDCs and iECDCs at passage 30 was analyzed. Briefly, ECDCs were incubated with 10 μg/ml nocodazole (Sigma-Aldrich, St.Louis, MO, USA) in fresh CEM for 6 h to enrich arrested metaphases, followed by treatment with 0.075 M potassium chloride (KCl) for 40 min at 37°C, then fixed with cold methanol:acetic acid (3:1) and spread onto a clean glass slide. Chromosome numbers were counted in individual cells with fluorescence microscope (Olympus FV1000, Olympus, Japan) after staining with 4′,6-diamidino-2-phenylindole (DAPI). Telomere length of pECDCs and iECDCs at passage 30 was estimated by quantitative fluorescence *in situ* hybridization. Slides were denatured and hybridized with 0.5 μg/ml Fluorescein isothiocyanate (FITC)-Tel (Panagene, Korea) and were counterstained with 0.5 μg/ml DAPI. Chromosomes and telomeres were imaged with fluorescence confocal microscope. Data were analyzed using the online software “Telometer: Software for Telomere Counting” (http://demarzolab.pathology.jhmi.edu/telometer/). For quantitative analysis of telomere length, at least 30 individual cells were measured.

### Flow Cytometry

Flow cytometry experiments were performed using flow cytometer (FACSCalibur, BD Biosciences). 1 × 10^6^ primary EpiCs (pEpiCs), pECDCs, and iECDCs at passage 30 were suspended in Dulbecco's Phosphate Buffered Saline (DPBS) and incubated with the following antibodies: CD105 (eBioscience, Waltham, MA, USA), CD31 (eBioscience, Waltham, MA, USA), CD45 (eBioscience, Waltham, MA, USA), KIT proto-oncogene receptor tyrosine kinase (c-kit) (eBioscience, Waltham, MA, USA), kinase insert domain receptor (Kdr) (eBioscience, Waltham, MA, USA), stem cell antigen-1 (Sca-1) (eBioscience, Waltham, MA, USA), and CD90 (eBioscience, Waltham, MA, USA) at a dilution of 1:500 for 1 h at 4°C and isotype-matched antibodies served as negative controls. Fluorescent compensation was performed using single labeled controls and all the measures were performed using FlowJo (version 7.6, Tree Star Incorporation, Ashland, Oregon, USA).

### Immunocytochemistry and Immunohistochemistry

For immunocytochemistry, cells seeded onto the glass were fixed with 4% Paraformaldehyde (PFA) for 30 min at 37°C, then permeabilized with 0.5% Triton X-100 (Sigma-Aldrich, St.Louis, MO, USA) for 30 min, and blocked with 5% Normal Goat Serum (NGS) (Invitrogen, Carlsbad, CA, USA) for 2 h at room temperature (RT). Subsequently, cells were incubated with primary antibodies including myocyte enhancer factor 2A (Mef2a) (1:200, Abcam, Waltham, MA, USA), Gata4 (1:100, Abcam, Waltham, MA, USA), Desmin (1:100, Abcam, Waltham, MA, USA), a-smooth muscle actin (a-SMA) (1:400, Sigma-Aldrich, St.Louis, MO, USA), CD90 (1:200, Proteintech, Rosemont, IL, USA), Wt1 (1:50, Abcam, Waltham, MA, USA), Tbx18 (1:100, Abcam, Waltham, MA, USA), ISL1 transcription factor (Isl-1) (1:100, Abcam, Waltham, MA, USA), troponin T2, cardiac (cTNT) (1:400, Invitrogen, Carlsbad, CA, USA), transcription factor 21 (Tcf21) (1:100, Affinity, Pottstown, PA, USA), and uroplakin 3B (Upk3b) (1:50, Novus, USA) at 4°C overnight and then incubated with goat antimouse Alexa-488/594, donkey antirabbit Alexa-594/488 secondary antibodies (Invitrogen, Carlsbad, CA, USA) at a dilution of 1:200 for 1 h at RT, followed by mounting with mounting medium containing DAPI (Vector Laboratories, Burlingame, CA, USA). For immunohistochemistry, 7 μm heart paraffin imbedded slides from iECDCs-transplanted hearts and frozen section of pECDCs-transplanted hearts were prepared, then blocked with 10% NGS for 2 h at RT, incubated with primary antibodies including Green fluorescent protein (GFP) rabbit antibody (1:100, Proteintech, Rosemont, IL, USA), GFP mouse antibody (1:100, Proteintech, Rosemont, IL, USA), a-SMA (1:400, Sigma-Aldrich, St.Louis, MO, USA), collagen type 3a1 (1:400, Proteintech, Rosemont, IL, USA), cTNT (1:200, Invitrogen, Carlsbad, CA, USA), and platelet/endothelial cell adhesion molecule 1 (PECAM) (1:100, Abcam, Waltham, MA, USA), then the slides were incubated with corresponding goat antimouse Alexa-488/594, donkey antirabbit Alexa-488/594 secondary antibodies (Invitrogen, Carlsbad, CA, USA) at a dilution of 1:200 for 1 h at RT, followed by washing with 0.1% PBST, and mounting with mounting medium containing DAPI.

### *In vitro* Differentiation

To induce differentiation, 2 × 10^5^ iECDCs at passage 30 were seeded in 12-well plate and cultured in CEM. Once the cells were confluent, change the medium with smooth muscle differentiation medium [IMDM, 5% FBS, 10 ng/ml platelet-derived growth factor-β platelet derived growth factor subunit B (PDGF-β) (PeproTech, Cranbury, NJ, USA), and 20 ng/ml transforming growth factor, beta 1 (TGF-β1) (R&D, Emeryville, CA, USA)]; 5 days later, smooth muscle differentiation was confirmed through a-SMA immunofluorescence. To induce cardiac differentiation, 2 × 10^5^ iECDCs at passage 30 were seeded onto the cover slides and incubate with cardiomyocyte induction medium [DMEM-low glucose, 2% FBS, and 10 μM 5-azacytidine (Sellecks, USA)] for 3 days and then change the medium with low glucose-DMEM supplemented with 2% FBS for another 2 weeks. Then, these cells were fixed with 4% PFA (Sigma-Aldrich, St.Louis, MO, USA), followed by cTNT and desmin immunofluorescence identification, images were acquired with Olympus FV1000 confocal microscope.

### *In vitro* Vasculogenic Potential

To induce tube formation, 80,000 iECDCs at passage 30 per well were plated on 24-well plates coated with Matrigel basement membrane matrix (BD Biosciences, San Diego, CA, USA). 50 ng/ml vascular endothelial growth facto (VEGF) (PeproTech, Cranbury, NJ, USA) treated Human Umbilical Vein Endothelial Cells (HUVECs) served as positive control. Tube formation analysis was performed after 6 h of incubation through phase-contrast images (Olympus FV1000, Olympus, Japan).

### Ribonucleic Acid Sequencing

Total RNA (3 μg/sample) extracted from pEpiCs, pECDCs, and iECDCs at passage 30, 10 μM SB431542-treated pECDCs-derived epithelial clone 1, and 10 μM 5-azacytidine-treated iECDCs at passage 30 were used as input material. Sequencing libraries were generated using the NEBNext^®^ Ultra™ RNA Library Prep Kit for Illumina^®^ (NEB, Ipswich, MA, USA) or oligo(dT) method from Beijing Genomics Institute (BGI) platform per the instructions of the manufacturer and index codes were added to attribute sequences to each sample. The clustering of the index-coded samples was performed on the cBot Cluster Generation System using the TruSeq PE Cluster Kit v3-cBot-HS (Illumina, San Diego, CA, USA) according to the instructions of the manufacturer. After cluster generation, the library preparations were sequenced on the Illumina HiSeq 2500 platform or the BGISeq-500 platform (BGI-Shenzhen, China) and 100 bp pair-end reads were generated. Differential expression analysis of two conditions/groups (two biological replicates per condition) was performed using the DESeq R package (1.10.1). DESeq provides statistical routines for determining differential expression in digital gene expression data using a model based on the negative binomial distribution. The resulting *p*-values were adjusted using the approach by Benjamini and Hochberg for controlling the false discovery rate. Genes with an adjusted *p*-value < 0.05 found by DESeq were assigned as differentially expressed. The Gene Ontology (GO) enrichment analysis of differentially expressed genes was implemented by the GOseq R package, in which gene length bias was corrected. The GO terms with corrected *p* < 0.05 were considered significantly enriched by differentially expressed genes. Clustered heatmaps were produced by Cluster 3.0 software. Original data were uploaded to the Gene Expression Omnibus database with accession number: GSE131765, GSE159640, and GSE189275.

### Myocardial Infarction and Cell Delivery

For *in-vivo* heart IR, *in-situ* open-chest heart model was carried out. Briefly, C57BL/6 mice (8–10 weeks) were anesthetized with 80 mg/kg sodium pentobarbital intraperitoneally, intubated through a tracheotomy, and ventilated with 3 cm H_2_O positive-end expiratory pressure. Adequacy of anesthesia was monitored using corneal and withdrawal reflexes. Ventilation frequency was kept at 110 breaths/min with tidal volume between 135 and 150 μl. The left anterior descending (LAD) coronary artery was surrounded by a 7-0 Prolene suture that was then passed through a small plastic tube. Ischemia was induced by tightening the tubing against the heart surface. After 30 min of ischemia, the plastic tube was removed for reperfusion. Sham control mice underwent the same surgical procedures without left coronary artery (LCA) ligation. Regional ischemia was confirmed by visual inspection of pale color of the myocardium and the elevation of ST segment on the ECG. For the cell injection, Dil-labeled pECDCs or GFP-labeled iECDCs at passage 30 (4 × 10^5^) were injected in a volume of 40 μl of PBS (20 μl at each of 2 sites bordering the infarct) with 40 μl of PBS as controls. 6 weeks later, the mice were sacrificed and the hearts were collected for further identification. The engraft efficiency was quantified through the ratio of GFP-positive nucleus to all the nucleus in the obtained photograph of the transplanted heart section and the differentiation ability was confirmed through costaining of GFP with other cardiovascular cell lineage markers. All the mouse work was carried out in the Animal Unit, Zheng Zhou University (Zhengzhou, China) according to procedures by the Institutional Animal Care and Use Committee.

### Cardiac Function Measurements

Cardiac function was assessed by echocardiography. Briefly, 6 weeks post-iECDCs injection, sham group, PBS-treated, and iECDCs-transplanted C57BL/6 mice were examined by echocardiography performed in monodimensional mode (M-mode) with a high-resolution transducer at a frequency of 30 MHz (Vevo 770, Visualsonics Incorporation, Toronto, Canada). Mice were anesthetized by 1.5% isoflurane in oxygen. Parasternal and short-axis two-dimensional images of left ventricle were acquired to determine the correct M-mode cursor positioning. The heart rate was determined from at least three consecutive RR intervals. The left ventricle M-mode trace was used to measure ejection fraction (EF), fractional shortening (FS), left ventricular internal dimension-diastole (LVIDd), LVID-systole (LVIDs), end-diastolic volume (EDV), and end-systolic volume (ESV).

### Exosome Isolation and Characterization

Cell culture medium from iECDCs and dC2C12 was firstly centrifuged at 1,000 g for 10 min, followed by 10,000 g for 30 min. The supernatant was collected and filtered through a 0.22-μm filter (Millipore, Billerica, MA, USA), followed by ultracentrifugation at 100,000 g for 1 h to pellet exosomes. Exosomes were resuspended in sterile PBS and packed and stored in −80°C for further testification. The size distribution of exosomes was measured by Nanoparticle Tracking Analysis (NTA) (Nanosight, Westborough, MA, USA). Transmission electron microscopy of exosomes was performed and imaged with a high-resolution transmission electron microscope (Hitachi HT7700, Japan) as previously described (34).

### Western Blot for Exosomes

The total protein concentration of exosomes was quantified using the Bradford assay (Sangon Biotech, Shanghai, China). A 30-μg protein was loaded to 10% sodium dodecyl sulfate (SDS)-polyacrylamide gel and electrophoresed for 2 h at 80 V, followed by transferred with polyvinylidene fluoride (PVDF) membranes for 2.5 h at 250 mA at 4°C. Membranes were blocked with 5% skimmed milk (BD Biosciences, San Diego, CA, USA) and probed with different primary antibodies including Alix (1:1,000, Cell Signaling Technology, Danvers, MA, USA), CD63 (1:1,000, Abcam, Waltham, MA, USA), CD81 (1:200, Santa Cruz Biotechnology, Dallas, Texas, USA), and CD82 (1:1,000, Abcam, Waltham, MA, USA). The bound primary antibody was then detected by peroxidase-conjugated rabbit antimouse or goat antirabbit immunoglobulins (IgGs) (1:5,000, Sigma-Aldrich, St.Louis, MO, USA), respectively, followed by developed through the enhanced chemiluminescence (ECL) Western Blotting Analysis System (Amersham Pharmacia Biosciences, Cambridge, UK).

### Masson's Trichrome Staining and TTC Staining

A total of 8 μm heart sections were fixed overnight in Bouin's solution followed by staining with the Masson's Trichrome Staining Kit (Solarbio, China) as per the instructions of the manufacturer. The images were captured with the Pannoramic DESK Digital Slide Scanner (3DHISTECH, Budapest, Hungary) and infarct area was measured by the ratio between the collagen-positive area and total area through (Image J software, Bethesda, MD, USA). For the Triphenyl tetrazolium chloride (TTC) staining, hearts were isolated and fixed in optimal cutting temperature (OCT) (Sakura Finetek Incorporation, Torrance, CA, USA) for 30 min at −20°C. Then, the heart was sectioned into 7 slices of 2 mm thickness from the base of the heart to the apex. The slides were stained at 37°C for 30 min with 0.5% triphenyltetrazolium chloride (Solarbio, China) in saline and the infarcted area exhibits white.

### Statistical Analysis

All the data are reported as means ± SEM. Statistical differences between different groups were evaluated by SigmaStat (Systat Software Incorporation, Chicago, Illinois, USA). Both the parametric and non-parametric analyses were applied as specified in figure legends. Significance was determined based in *p* < 0.05.

## Results

### Primary Epicardium-Derived CDCs Differs From pEpiCs, While Highly Expressed Tbx18 Transcriptional Factor

**Figure 1:**
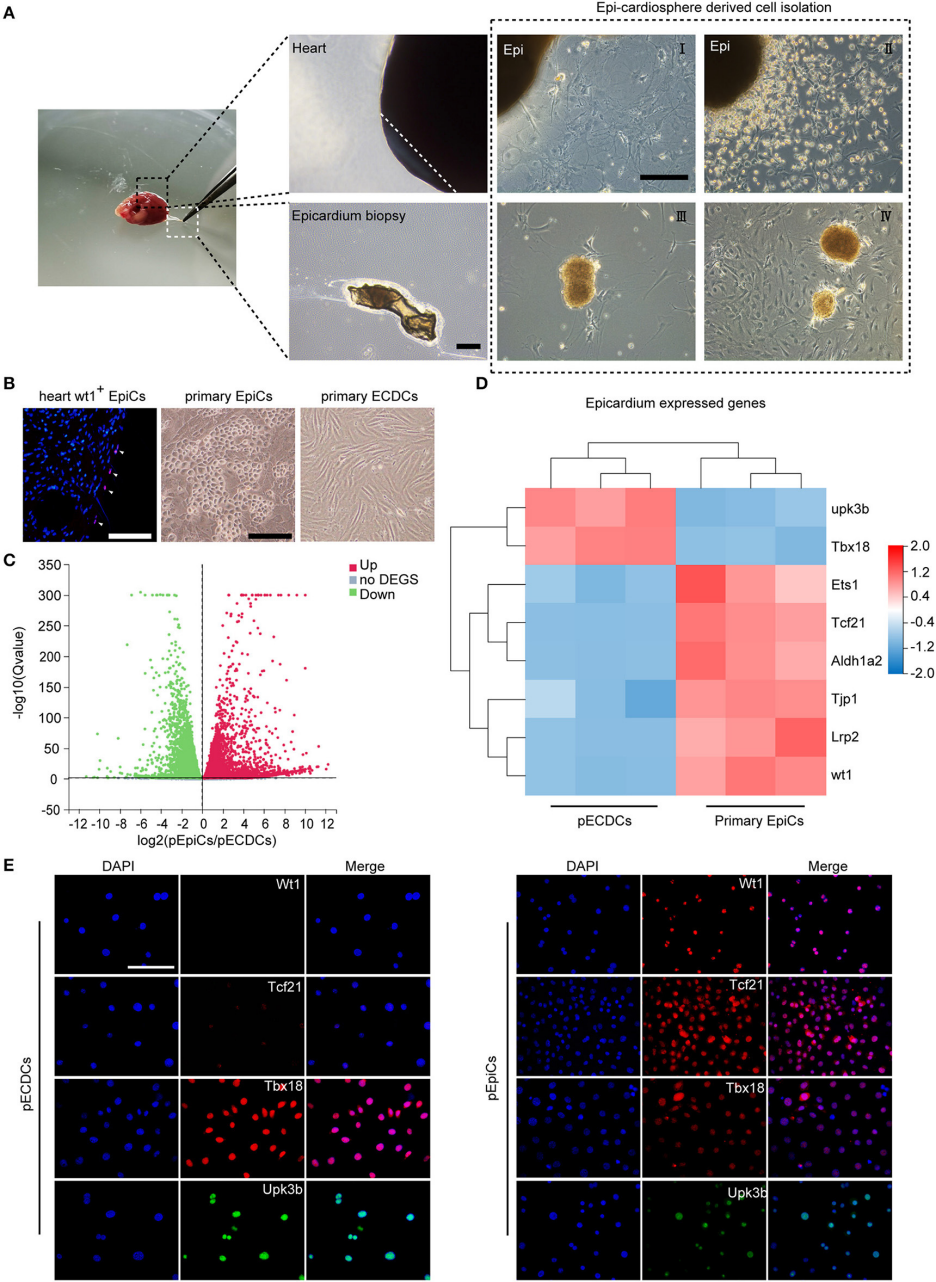
Isolation of primary epicardium cardiosphere-derived cells (pECDCs) and gene expression profile analysis compared with primary epicardial cells (pEpiCs). **(A)** Isolation and culture of pECDCs from adult mouse ventricle epicardium (scale bar = 100 μm). **(B)** Heart Wt1^+^ EpiCs staining and bright morphology of isolated primary pECDCs and pEpiCs (scale bar = 100 μm). **(C)** Differential gene expression volcano plot of RNA sequencing results of pECDCs and pEpiCs. Data represent mean ± SEM (*n* = 3). **(D)** Heatmap analysis of epicardium-related genes to compare pECDCs with pEpiCs. Expression levels were identified with different colors in which red represents upregulation and green represents downregulation. **(E)** Immunostaining of T-box transcription factor 18 (Tbx18) and other epicardium markers of pECDCs and pEpiCs (scale bar = 100 μm).

The epicardium is a thin, transparent epithelial layer enveloping the outside of the heart. Based on the epicardium isolation protocol previously described (32), the epicardium was directly peeled off from the 6-week aged C57 mouse heart. To isolate CDCs, we applied the four-stage classical method reported by Smith et al. (2) (Figure 1A). Briefly, the epicardium-derived small, round, and phase-bright cells were collected and seeded onto poly-D-lysine-coated dishes. CDCs-formed cardiospheres detached from the bottom on day 30 post-seeding. Replating of cardiospheres resulted in the monolayer of pECDCs. EpiCs specifically express Tbx18, transcription factor 21 (Tcf21), Wilms' tumor suppressor transcription factor (Wt1), uroplakin 3B (Upk3b), and other identified metabolism-related genes from single cell sequencing results (35, 36). EpiCs located in the heart epicardium from Wt1 immunofluorescence and pEpiCs isolated from the adult heart epicardium exhibited epithelial cell morphology, which differed from mesenchymal cell morphology of pECDCs (Figure 1B). Flow cytometry assay (Figure 3B, Supplementary Figure S2) demonstrated that pECDCs uniformly express reported CDC-specific surface markers, transforming growth factor-beta receptor accessory subunit, CD105 (endoglin), and glycosylphosphatidylinositol (GPI)-anchored protein CD90 (Thy-1), while pEpiCs were mostly negative for CD90. Meanwhile, both the pECDCs and pEpiCs expressed the stem cell-associated marker such as Sca-1 and were negative for stem cell-associated marker such as c-kit as well as hematopoietic cell and endothelial cell markers such as Kdr, CD31, and CD45. Due to the epicardium origin of ECDCs, we explored the relationship between pECDCs and pEpiCs through RNA sequencing. Gene expression volcano Plot results showed a big difference between pECDCs and pEpiCs (Figure 1C), especially for epicardium-specific genes expression, which was confirmed from immunostaining (Figures 1D,E). Interestingly, despite the lack of Wt1 and Tcf21 expression, pECDCs highly expressed Tbx18 and Upk3b (Figure 1D), suggesting close relationship between ECDCs and EpiCs. Considering reduced expression of epithelial cell marker-tight junction protein 1 (Tjp1), we deduced that ECDCs may derive from EpiCs EMT and retain part of EpiCs characters.

### Human Telomerase Reverse Transcriptase-Transduced ECDCs (iECDCs) Possess Self-Renewal Capacity Without Chromosomal Alteration and Telomere Shortening

**Figure 2:**
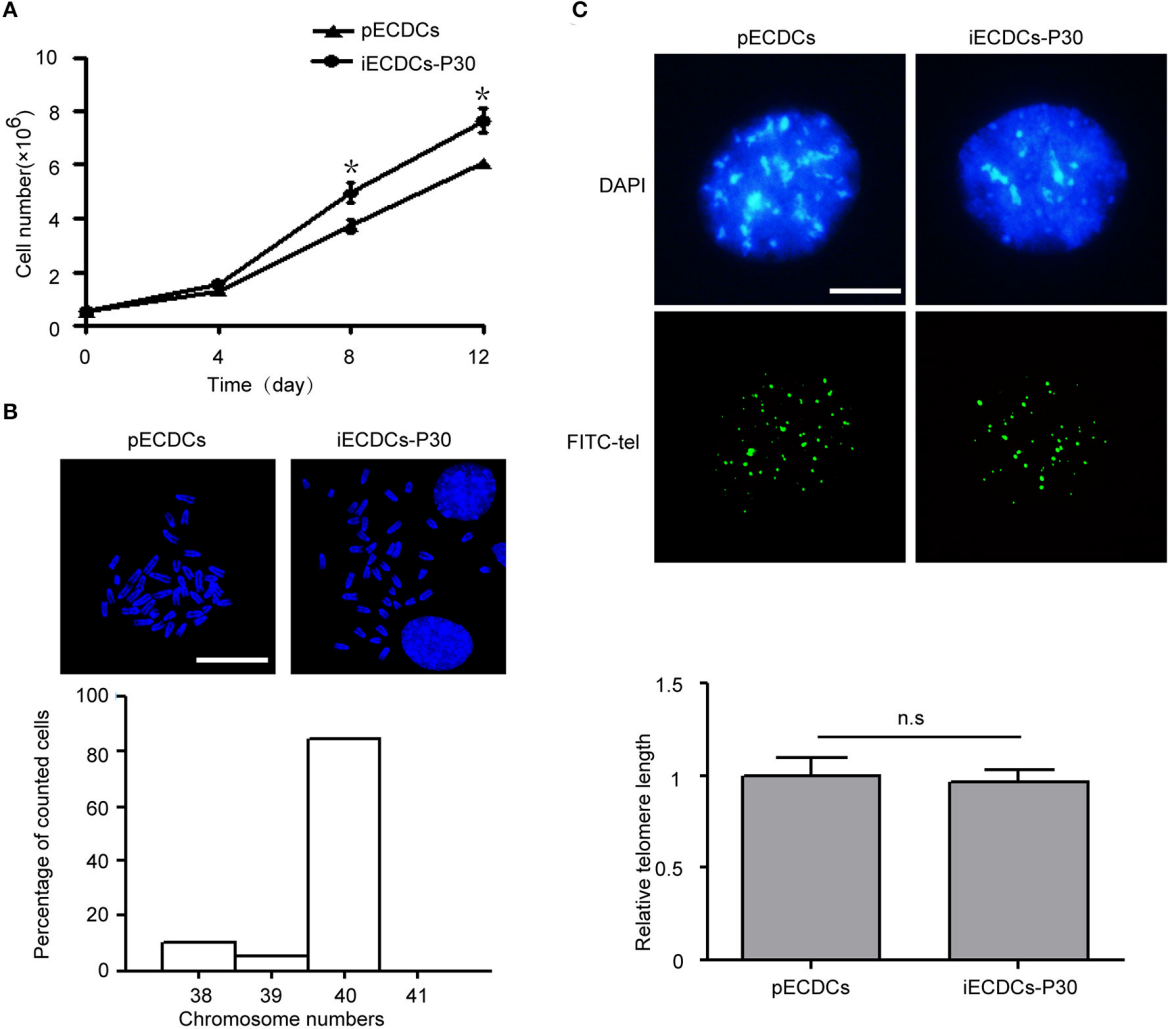
Establishment of mouse immortalized ECDCs (iECDCs). **(A)** Growth curves of isolated pECDCs and iECDCs at passage 30. Data represent mean ± SEM (*n* = 3, *p < 0.05, two-tailed *t*-test). **(B)** 4′,6-diamidino-2-phenylindole (DAPI) staining and quantity statistics of chromosome numbers during mitosis in iECDCs at passage 30 (*n* = 25). **(C)** Telomere Quantitative Fluorescent *in situ* hybridization (Q-FISH) and quantification of pECDCs and iECDCs at passage 30 (*n* = 30, ns means no significant difference, two-tailed *t*-test).

Primary epicardium-derived CDCs can be propagated for passaged 10 generations and then experience cell differentiation and death. hTERT has been used to immortalize multiple kind of primary cells (37). In order to acquire enough cells for further studies, we packaged hTERT lentivirus and infected pECDCs, following by puromycin selection. After transduction, iECDCs exhibit a continuous proliferation ability during about 1 year of culture and passage (Figure 2A) and little difference was detected in the doubling times between passages 30 and 80 (data not shown), suggesting that iECDCs retained self-renewal capacity after hTERT infection. Consistently, the Kyoto Encyclopedia of Genes and Genomes (KEGG) pathway analysis demonstrated the significant upregulation of DNA replication- and cell cycle-related genes in iECDCs compared with pECDCs (Supplementary Figures S1a,b). To exclude immortalization caused by chromosomal abnormalities, we did karyotype analysis and the results showed that about 80% of karyotypes have 40 chromosomes (Figure 2B), without telomere shortening (Figure 2C).

### Immortalized ECDCs Showed a Cardiac-Specific Gene Expression Pattern

**Figure 3:**
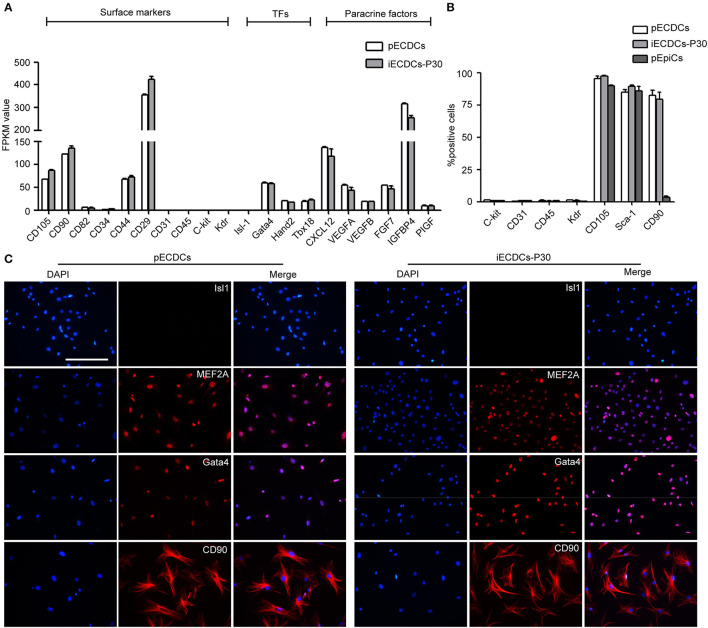
Lineage specification of pECDCs and iECDCs. **(A)** The expression of surface markers, transcription factors, and paracrine factor genes of pECDCs and iECDCs. The pECDCs and iECDCs at passage 30 were used for RNA sequencing. Fragments Kilobase of exon model permillon mapped reads (FPKM) values were used for quantification of gene expression level. Data represent mean ± SEM (*n* = 3). **(B)** Flow cytometric analysis of cell surface markers of pECDCs, pEpiCs, and iECDCs at passage 30, using anti-c-kit, CD31, CD45, Kdr, CD105, CD90, and stem cell antigen-1 (Sca-1) antibodies. Data represent mean ± SEM (*n* = 3). **(C)** Immunostaining of CDC cell markers for pECDCs and iECDCs (scale bar = 100 μm).

To further characterize pECDCs and iECDCs, we performed the RNA sequencing to compare gene expression profiles of pECDCs and iECDCs (passage 30). The results showed that iECDCs retained the similar surface antigen expression pattern to pECDCs including CD105, CD90, Sca-1, CD31, CD45, Kdr, and c-kit (Figure 3A). Flow cytometry assay confirmed the expressions of these markers on iECDCs and no significant difference was observed between pECDCs and iECDCs (Figure 3B). Besides, we observed the expression of cardiac-specific transcription factors in iECDCs such as Gata4 and Hand2. iECDCs do not express the second-field heart progenitor cell marker such as Isl1, confirmed via immunofluorescence images (Figures 3A,C). Paracrine factors, which are regarded as key regulator benefitting CDCs therapeutic effect in heart diseases, were highly expressed in iECDCs [chemokine (C-X-C motif) ligand 12 (CXCL12), VEGF, FGF, IGFBP4, etc.] (Figure 3A). Based on these results, iECDCs retained most of CDCs gene expression pattern, similar to pECDCs, indicating that hTERT induction could be used for pECDCs expansion and iECDCs could be utilized as a cell model for further functional study.

### Immortalized ECDCs Have a Multilineage Differentiation Potential

**Figure 4:**
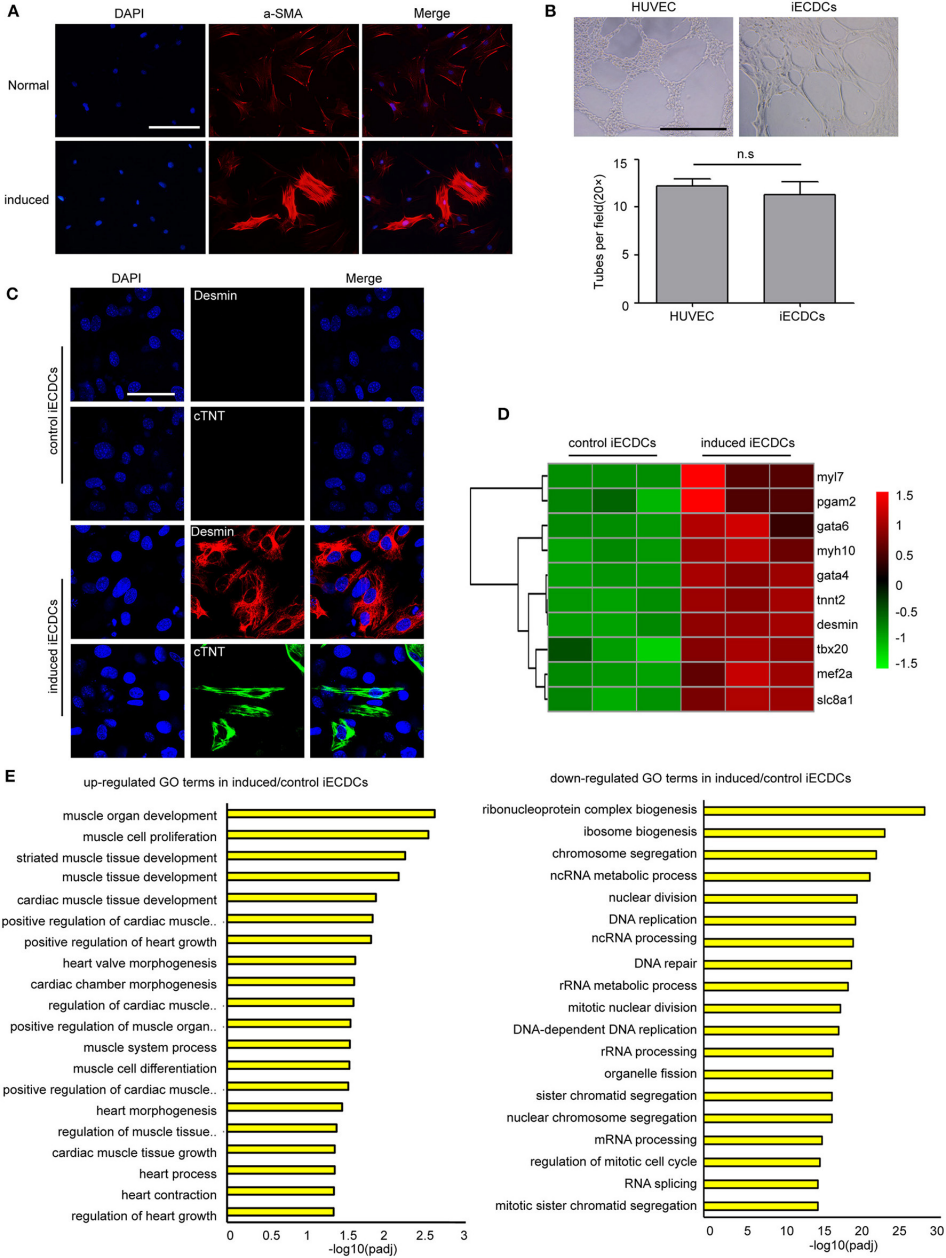
Cardiovascular differentiation potential of iECDCs *in vitro*. **(A)** Immunostaining of iECDCs with a-SMA pre- and postsmooth muscle differentiation induced by TGF-β1 and PDGF-β (scale bar = 100 μm). **(B)** Analysis of blood vessel network formation after 6 h culture on Matrigel of HUVEC and iECSCs (*n* = 8, two-tailed *t*-test). **(C)** Confocal imaging of cardiac marker proteins desmin and cTNT in induced iECDCs (scale bar = 100 μm). **(D)** Heatmap analysis of cardiomyocyte-related genes to compare induced iECDCs with normal iECDCs. Expression levels were identified with different colors in which red represents upregulation and green represents downregulation. **(E)** The Gene ontology (GO) analysis results up- and downregulated GO terms in induced iECDCs vs. normal iECDCs based on the RNA sequencing assay (*n* = 3, *p < 0.05, two-tailed *t*-test).

Tbx18^+^ EpiCs give rise to multiple cardiac cells during heart development including myocytes, fibroblasts, and smooth muscle cells (31). To determine whether Tbx18^+^ iECDCs possess stem cell properties, we analyzed their ability to differentiate into three major heart cell lineages: smooth muscle cells, endothelial cells, and cardiomyocytes. iECDCs differentiated into a-SMA^+^ smooth muscle cells (61.41 ± 3.22%) (Figure 4A), desmin^+^ myocytes (21.88 ± 4.85%), and limited cTNT^+^ myocytes (3.52 ± 1.1%) (Figure 4C), indicating that cardiac differentiation mainly produced immature cardiomyocytes (Desmin^+^/cTNT^−^). The GO analysis demonstrated activation of the cardiac muscle development process and inhibition of DNA replication after treatment of 5-azacytidine (Figure 4D). Consistently, heatmap analysis showed a remarkable increase of cardiomyocyte-specific gene expression in the induced-iECDCs group (Figure 4E). Tube-formation assay is one of the simple, but well-established *in-vitro* angiogenesis assays. iECDCs formed efficiently tube-like networks after 6 h culture on matrigel (Figure 4B), suggesting its vasculogenic and angiogenic potential of iECDCs. These data confirmed the cardiovascular differentiation ability of iECDCs *in vitro*.

### Immortalized ECDCs Transplantation Decreased Fibrosis and Improved Heart Function in MI Mouse Model

**Figure 5:**
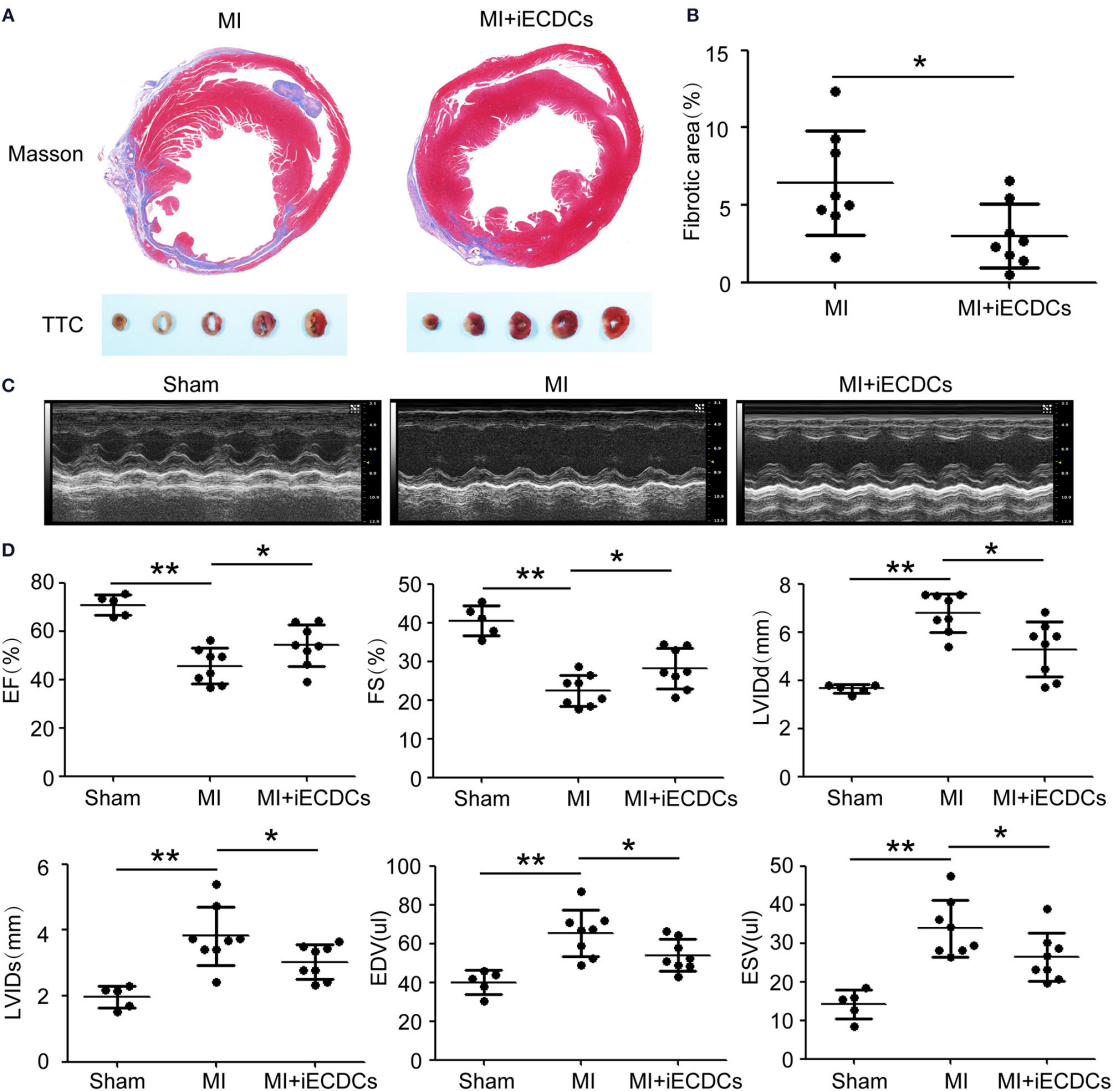
iECDCs graft improved cardiac function *in vivo*. **(A)** Representative images of Masson's trichrome staining and TTC staining for iECDCs-transplanted and control heart injected with same volume of phosphate-buffered saline (PBS) at 6 weeks postmyocardial infarction (MI) (scale bar = 500 μm). **(B)** Quantification of the fibrotic area in iECDCs-transplanted hearts vs. PBS-treated hearts at 6 weeks post-MI (*n* = 8, *p < 0.05, **p < 0.01, one-way ANOVA *post-hoc* student Newman–Keuls test). **(C)** Representative echocardiographic images in different groups. **(D)** Cardiac function were evaluated by echocardiography (*n* = 8, *p < 0.05, one-way ANOVA *post-hoc* student Newman–Keuls test). Data are presented as mean ± SEM.

It has been previously reported that CDCs transplantation preserved cardiac function in multiple injury animal models (18–20). Whether epicardium-derived Tbx18^+^ CDCs have the same effect, it has not been demonstrated. To explore the infusion and function of iECDCs in the heart of the MI mouse, GFP-labeled iECDCs (Supplementary Figure S3a) (4 × 10^5^) were injected in 2 sites bordering the infarct and PBS-injected mice were regarded as controls. 6 weeks later, the mice were sacrificed and the hearts were collected for further identification. Echocardiography was utilized to evaluate heart function. 6 weeks post-MI, infarcted areas, especially in the left ventricle, were observed apparently in PBS-treated mouse (6.61 ± 1.4%), while significantly decreased in the iECDCs transplanted group (3.01 ± 0.8%) (Figures 5A,B). TTC staining was in line with the Masson's trichrome staining results (Figure 5A). Figures 5C,D shows improvement of left ventricle ejection fraction (LVEF) in the iECDCs transplanted group (54.2 ± 3.5%) compared to the PBS-treated group (45.08 ± 2.9%). Consistently, FS values increased in the transplanted group (28.29 ± 2.1%, *p* < 0.05) compared to the PBS-treated group (22.21 ± 1.6%, *p* < 0.05). Moreover, the LVIDd was reduced in the transplanted group (5.19 ± 0.5 mm) vs. the PBS-treated group (6.84 ±0.3 mm, *p* < 0.05). The sham group exhibited normal heart function, LVEF (70.33 ± 2.4%), FS (40.4 ± 2.3%), and LVIDd (3.64 ± 0.1 mm) (Figures 5C,D). The data indicated that ECDCs graft prominently inhibited fibrosis and improved cardiac function in the MI mouse model.

### Immortalized ECDCs Differentiated Into Cardiomyocytes and Smooth Muscle Cells in the Mouse Model

**Figure 6:**
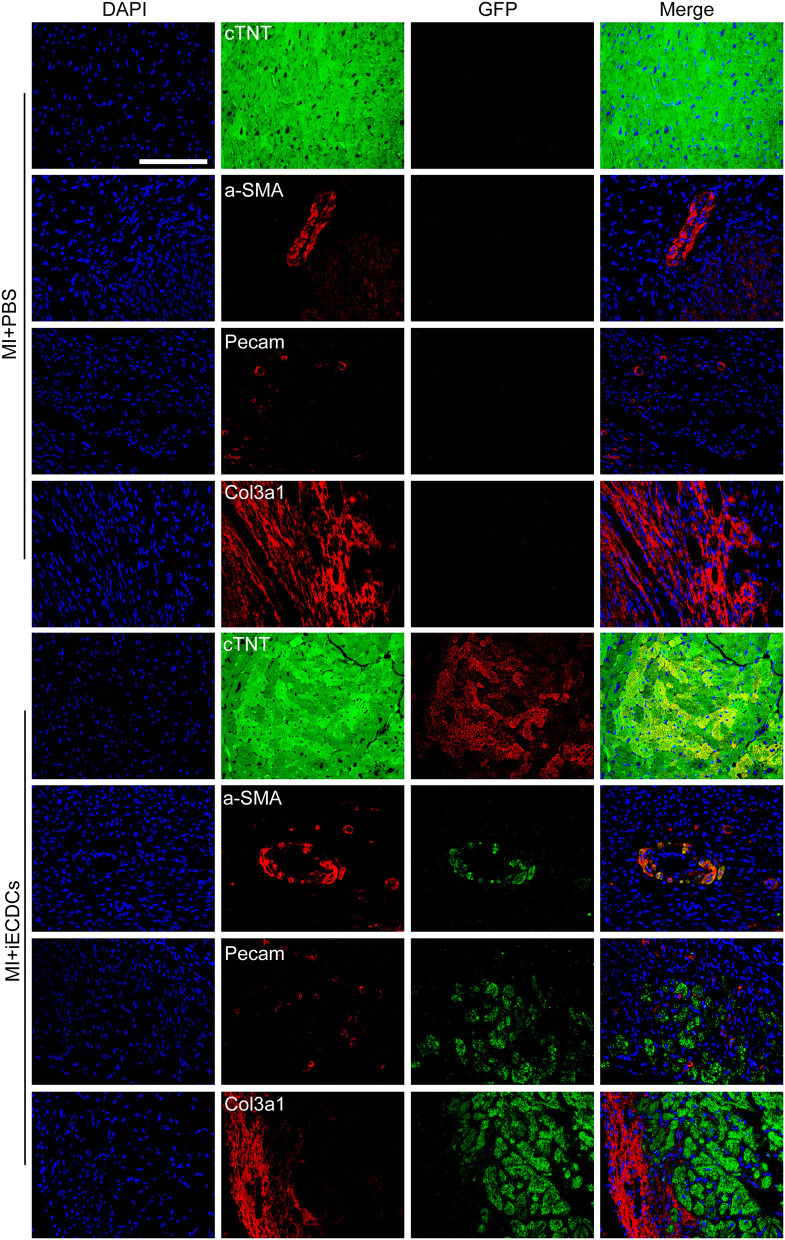
iECDCs differentiated into cardiomyocytes and smooth muscle cells in the mouse model. GFP^+^ iECDCs-transplanted hearts were harvested and sectioned 6 weeks post-MI. Representative images of GFP costained with cTNT, a-SMA, PECAM, and Col3a1, which indicated cardiomyocyte, smooth muscle cell, endothelial cell, and fibroblast, respectively (scale bar = 100 μm).

To verify the multilineage differentiation ability of iECDCs in the heart of the MI mouse model, we performed the counterstaining between GFP and different cardiovascular lineage markers (cTNT, a-SMA, PECAM, and Col3a1) to detect the iECDCs graft *in vivo*. GFP immunofluorescence results demonstrated successful integration of transplanted iECDCs in the mouse heart (7.35 ± 4.7%) (Supplementary Figure S3b). Unlike other reported studies *in vivo*, interestingly, these cells mainly differentiated into cTNT-positive cardiomyocytes instead of endothelial cells or fibroblasts (Figure 6 and Supplementary Figure S3c) and only several GFP/a-SMA double-positive vessels were observed in the transplanted myocardium around the injury site (Figure 6), indicating that the necrosis and inflammation released factors may affect transplanted iECDCs differentiation. In contrast, Dil-labeled pECDCs do not differentiate into cardiomyocytes, endothelial cells, or smooth muscle cells in the transplanted MI mouse heart; on the contrary, these cells colocalized with extracellular matrix (ECM)-Col3a1 and seem to be in the stage of apoptosis (Supplementary Figure S4), suggesting the potential of telomere homeostasis in improving the survival efficiency and differentiation ability of ECDCs transplantation in the mouse heart.

### Characterization of iECDCs-Derived Exosomes

**Figure 7:**
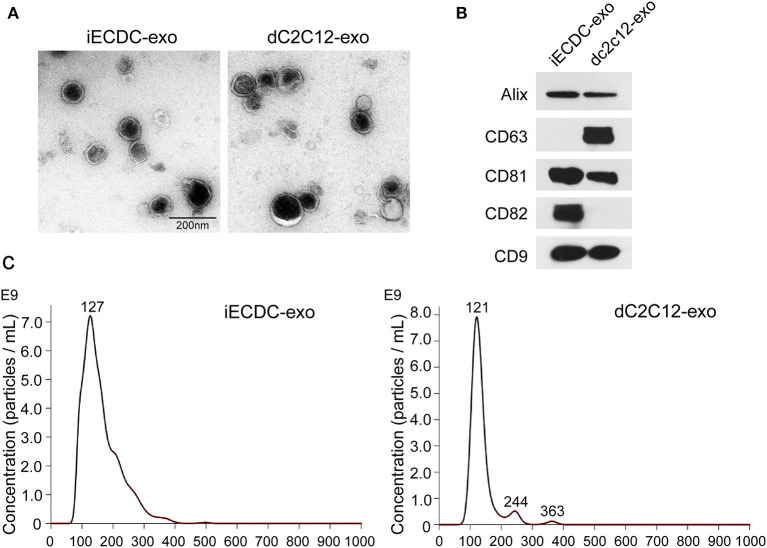
Characterization of iECDC exosomes. **(A)** Transmission electron microscope images of isolated dC2C12 and iECDC exosomes (scale bar = 200 nm). **(B)** Western blots to detect membrane proteins on dC2C12 and iECDC exosomes. Total protein (30 μg) was loaded and anti-Alix, CD63, CD81, CD82, and CD9 antibodies were used. **(C)** Measurement of exosome size distribution with Nanoparticle Tracking Analysis (NTA) (Nanosight, Westborough, MA, USA).

Exosomes are vesicles secreted by many types of cells, containing abundant small RNAs and proteins, which mediate cell-to-cell communication (38). Recently, CDC exosomes (CDC_exo_) were considered beneficial paracrine factors, contributing to cardioprotection in CDC-based cell therapy in cardiovascular diseases (39). To characterize iECDCs-derived exosomes (iECDC_exo_), we detected the expression of exosome surface markers. Western blot results showed a distinct expression pattern of membrane proteins between iECDC_exo_ and differentiated C2C12-derived exosomes (dC2C12_exo_). Compared to dC2C12_exo_ carrying more CD63, iECDC_exo_ highly expressed CD82, which was an ideal cargo for peptide loading (Figure 7B). As expected, iECDC_exo_ exhibited a classical bilayer membrane structure under transmission electron microscopy (Figure 7A). Simultaneously, NTA revealed that the average vesicle size of iECDC_exo_ was 127 nm in diameter, similar to 121 nm of the dC2C12_exo_ (Figure 7C). It suggested that iECDCs could yield functional exosomes with therapeutic potential.

## Discussion

Cardiosphere-derived cells are now considered as one of the most promising progenitor cells for clinical application (40). Mechanistic studies showed that CDCs graft robustly improved cardiac function, decreased scar size, and promoted myocardium survival, especially in acute myocarditis (7, 8). However, CDCs are derived from heart biopsy consisting of heterogeneous cell subpopulations, leading to an indistinct understanding of origin of CDCs. Epicardium is an evolutionarily conserved layer covering the outside of the heart. During cardiac development and injury, EpiCs underwent EMT, migrate from the epicardium, served as a progenitor cell pool, and principally give rise to fibroblasts and vascular smooth muscle cells verified from lineage tracing *in vivo* (21–24).

To investigate whether epicardium could yield CDCs, we directly isolated the epicardium of the adult mouse heart. According to referred methods, epicardium-derived cells also form cardiospheres on a poly-D-lysine-coated dish, while requiring a longer time than other groups reported. As expected, ECDCs express well-characterized CDCs membrane markers, CD105, CD90, and Sca-1, as well as cardiac stem cell transcriptional factors, Gata4 and Mef2a. Primary EpiCs highly express Wt1, Tcf21, and Tbx18, while ECDCs retained the expression of Tbx18 and Upk3b and were negative for Wt1 and Tcf21. Although RNA sequencing results showed a differed gene expression profile, epicardial explant-derived pECDCs may originate from EpiCs EMT, which could be triggered by TGF-β factors (41). To investigate the relationship between pECDCs and pEpiCs, we used an effective TGF-β signaling pathway inhibitor-SB431542 to inhibit or reverse the EMT process produced in pECDCs culture. Ten days post-treatment, we found several epithelial clones formed, which re-expressed Wt1 and Tcf21, indicating close relationship between ECDCs and EpiCs EMT (data not shown).

To prevent the induced cell death and lineage variation during long-time culture, we used hTERT to immortalize primary ECDCs. After extensive testification, we verified that hTERT iECDCs have a better proliferation capacity without change of gene expression profile or chromosome aberration. Moreover, iECDCs have multipotentiality to cardiomyocytes, smooth muscle cells, and vasculogenic cells *in vitro*. Interestingly, inconsistency between desmin and cTNT staining indicated that 5-azacytidine induction may give rise to immature cardiomyocytes.

Cardiosphere-derived cells transplantation elicits cardioprotection function that has been elucidated in different animal models with MI injury (9, 42, 43). We showed that MI mice exhibited severe cardiac dysfunction with cardiomyocyte necrosis, fibrosis, and thinning of the ventricular wall, while iECDCs transplanted hearts displayed better contractility and reduced fibrosis. Furthermore, despite the reported poor survival efficiency of CDCs in infarcted myocardium (44), we did detect the transplanted GFP-positive iECDCs in the hearts of recipients. Strikingly, the labeled iECDCs mainly differentiated into cTNT-positive cardiomyocytes, instead of endothelial cells and fibroblasts, and only a few vessels were double positive for a-SMA and GFP. Nevertheless, limited differentiation ability of Dil-labeled primary ECDCs was observed in transplanted mouse heart (Supplementary Figure S4). Telomere shortening was closely relevant to stem cell senescence and differentiation (45–47), so that hTERT expression may be responsible for the difference between pECDCs and iECDCs engraft and differentiation ability *in vivo*.

Different differentiation abilities between *in vitro* and *in vivo* may result from paracrine regulation enhanced by cardiac injury because smooth muscle differentiation mainly exists in the myocardium close to the injury site. One limitation of this study was that iECDCs transplantation sites were different within the group. Although iECDCs could survive in the recipient myocardium, we cannot exclude that the distance between injection sites and ligation sites may affect the differentiation and cardiac functional rescue *in vivo*. Simultaneously, RNA sequencing results showed various growth factors expression in iECDCs, which implied that paracrine regulation might also benefit iECDCs transplantation-induced heart protection. Another raised concern in this study was about whether Tbx18^+^ EpiCs contribute to cardiomyocyte in fetal or adult hearts. In the embryonic heart, Tbx18 was not only expressed in the epicardium, but also in the cardiomyocytes residing in the interventricular septum and the left ventricle (48), thus lineages tracing studies with Tbx18-cre in mice could not prove the cardiac differentiation capacity of Tbx18^+^ EpiCs. Though Tbx18^+^ iECDCs possess the cardiomyocyte differentiation potency, we could not conclude that Tbx18^+^ EpiCs could differentiate into cardiomyocytes in terms of heart development. Meanwhile, whether Tbx18^+^ ECDCs exist in the heart epicardium or *in-vitro* culture produced cell pool was still a challenging issue.

Accumulating evidence indicates CDC secreted exosomes as a key element that mediates indirect and long-lasting cardioprotection in CDC transplantation. Systemic CDC_exo_ administration seems to be a candidate substitute for CDC cell therapy in multiple cardiovascular diseases (8, 11, 49, 50). In this study, we characterized iECDC_exo_ and found that it exhibits a different membrane protein expression pattern than dC2C12_exo_. iECDC_exo_ highly expressed CD82, while dC2C12_exo_ highly expressed CD63. Gao et al. (34) reported an anchor peptide CP05, specifically binding to CD63, enriched on the exosome surface. Murine myotube C2C12 exosomes modified with CP05 and phosphorodiamidate morpholino oligomers (PMO) administration successfully rescued dystrophin expression and improved skeletal muscle function in the DMD mouse model. Phage screening of CD82-binding peptides might provide a tool for ECDC_exo_ engineering with DNA, RNA, and peptides, thus functioning as cargo for targeted therapeutic drug delivery in dystrophic cardiomyopathy and other cardiovascular injuries.

In summary, we isolated and established an immortalized ECDC cell line with a cardiac progenitor cell-related gene expression pattern, multipotentiality, and cardioprotection capacity in the MI mouse model. Our studies revealed that functional CDCs could yield from the epicardium. Meanwhile, ECDCs and ECDC_exo_ may be a promising model to investigate CDC cell therapy and exosome therapy.

## Data Availability Statement

The datasets presented in this study can be found in online repositories. The names of the repository/repositories and accession number(s) can be found in the article/Supplementary Material.

## Ethics Statement

The animal study was reviewed and approved by the Institutional Animal Care And Use Committee of Zheng Zhou University.

## Author Contributions

ZG designed the project. ZG and MG performed most of the experiments. LQ helped with the transcriptome analysis and cell transplantation. BH and SL wrote the manuscript. All authors contributed to the article and approved the submitted version.

## Funding

This study is supported by the National Natural Science Foundation of China (Grant No. 31970836 and 81801549) and the National Health Commission Key Laboratory of Birth Defects Prevention (Grant No. ZD202003).

## Conflict of Interest

The authors declare that the research was conducted in the absence of any commercial or financial relationships that could be construed as a potential conflict of interest.

## Publisher's Note

All claims expressed in this article are solely those of the authors and do not necessarily represent those of their affiliated organizations, or those of the publisher, the editors and the reviewers. Any product that may be evaluated in this article, or claim that may be made by its manufacturer, is not guaranteed or endorsed by the publisher.
