# Supplementary material for: Epicardium-Derived Tbx18+ CDCs Transplantation Improve Heart Function in Infarcted Mice
Source: Front Cardiovasc Med. 2022 Jan 24;8:744353. doi: 10.3389/fcvm.2021.744353 (PMC8820322; doi:10.3389/fcvm.2021.744353)
Supplement: Supplementary file 1 [file Data_Sheet_1.docx]

**Supplemental Figures**

**Figure S1, related to Figure 2**


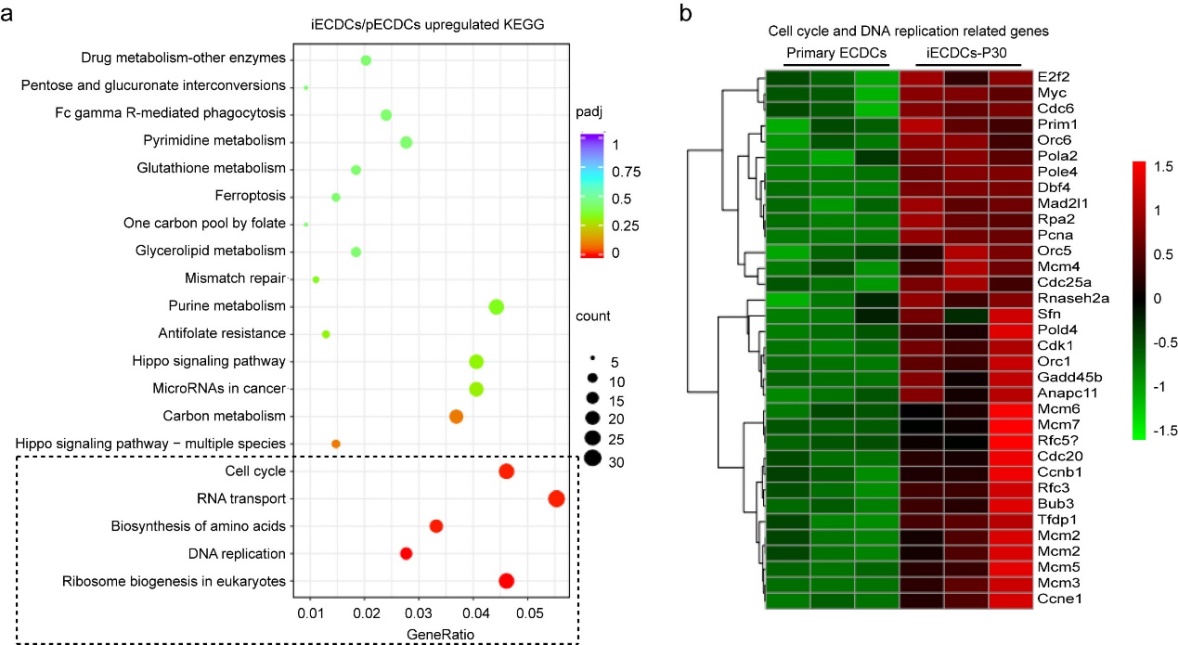


**Figure S1. Differential gene expression between primary ECDCs and immortalized ECDCs**. **a)** Up-regulated KEGG pathways in iECDCs versus pECDCs based on the RNA-sequencing assay (n=3, *p<0.05, two-tailed t test). **b)** Heatmap analysis of DNA replication and cell cycle-related genes to compare induced iECDCs with pECDCs. Expression levels were identificated with different colors in which red represents upregulation and green means downregulation (n=3, *p<0.05, two-tailed t test).

**Figure S2, related to Figure 3**


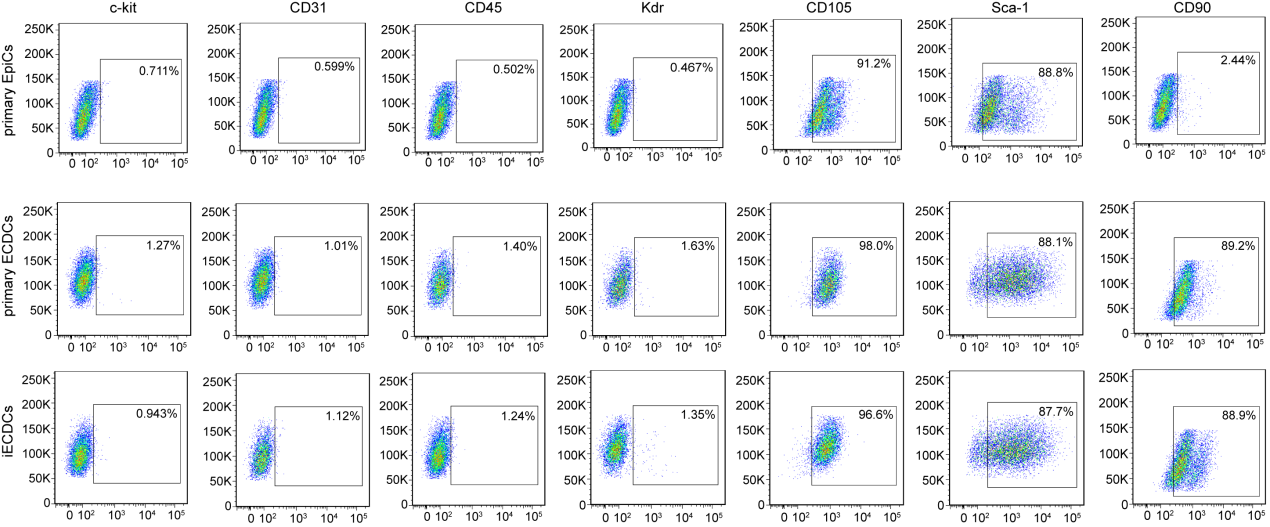


**Figure S2. Characterization of surface marker expression in iECDCs**. Flow cytometric analysis of c-kit,CD31,CD45,Kdr,CD105,CD90 and Sca-1 expression in primary EpiCs, ECDCs and iECDCs at passage 30.

**Figure S3, related to Figure 6**


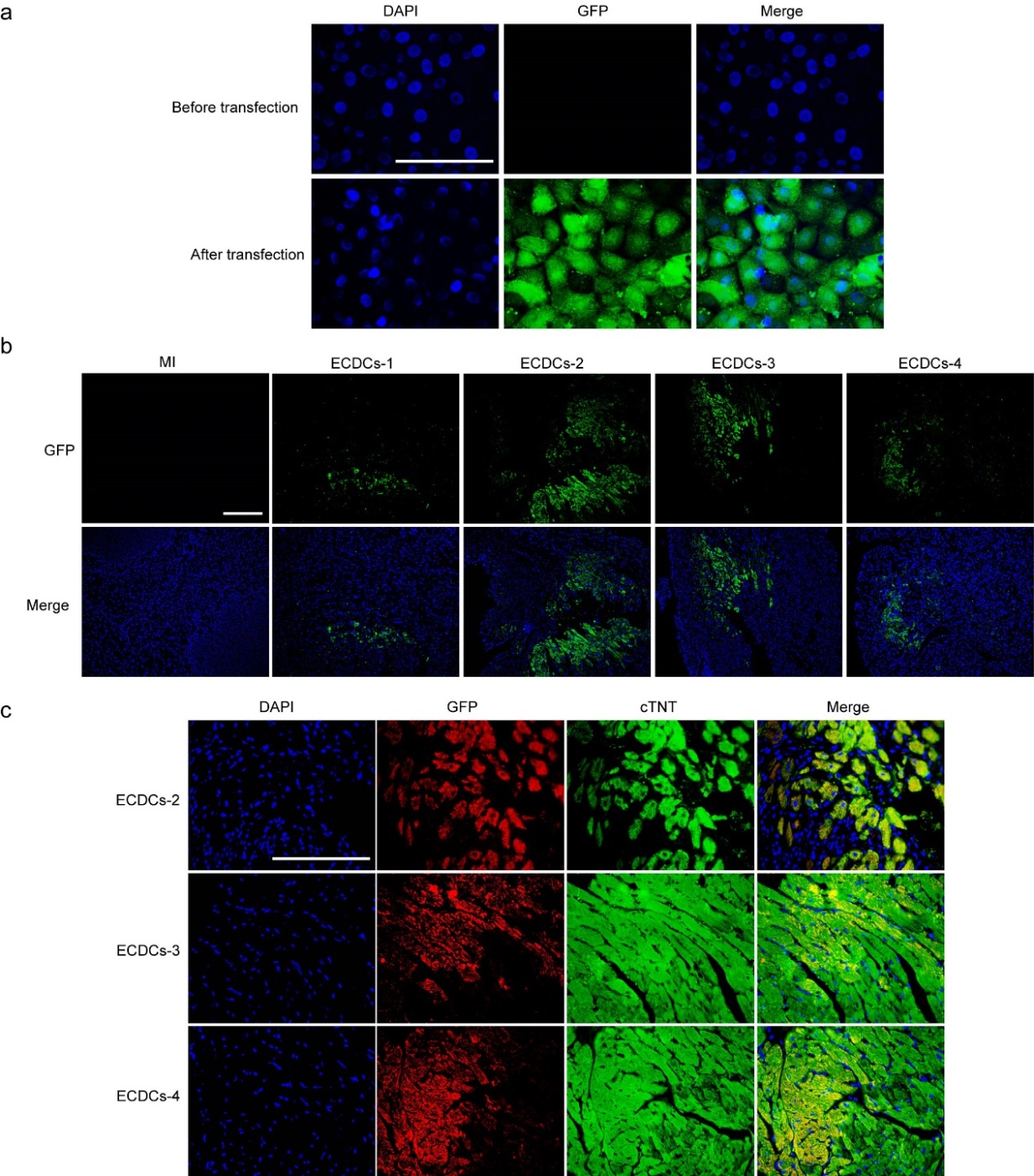


**Figure S3. iECDCs homing and differentiation in mouse heart**. **a)** Microscrope image of iECDCs transducted with GFP lentivirus (scale bar=100 μm). **b)** Immunostaining of GFP in the myocardium of iECDCs transplanted mouse heart under low magnification (scale bar=100 μm). **c)** Immunocytochemistry of GFP and cTNT in the myocardium of iECDCs transplanted mouse heart (scale bar=100 μm).

**Figure S4, related to Figure 6**


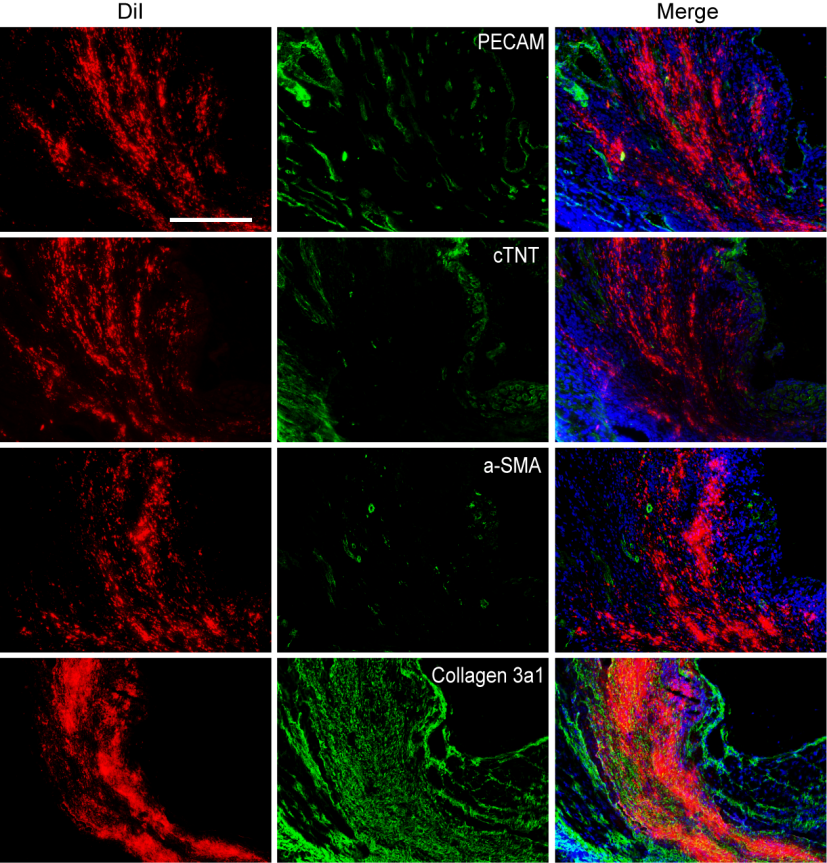


**Figure S4. pECDCs engraft and differentiation in mouse heart**. Co-staining microscrope image of Dil tagged primary ECDCs with different cardiovascular lineage markers (scale bar=100 μm).
